# The Role of Dietary Intake in the Weight Loss Outcomes of Roux-en-Y Gastric Bypass and Sleeve Gastrectomy: A Systematic Review and Meta-analysis

**DOI:** 10.1007/s11695-024-07183-8

**Published:** 2024-06-22

**Authors:** Dalal Qanaq, Majella O’Keeffe, Simone Cremona, Wanderley Marques Bernardo, Robert D. McIntyre, Efstathia Papada, Saumit Benkalkar, Francesco Rubino

**Affiliations:** 1 School of Cardiovascular and Metabolic Medicine and Sciences, King’s College London, James Black Centre, Denmark Hill Campus, 125 Coldharbour Road, London, SE5 9RJ UK; 2 College of Applied Medical Sciences, King Saud Bin Abdulaziz University for Health Sciences, 11481 Riyadh, Kingdom of Saudi Arabia; 3 King Abdullah International Medical Research Centre, 11481 Riyadh, Kingdom of Saudi Arabia; 4 Department of Nutritional Sciences, King’s College London, Franklin-Wilkins Building, 150 Stamford Street, London, SE1 9NH UK; 5 School of Food and Nutritional Sciences, University College Cork, College Road, Cork, Ireland; 6 Bariatric and Metabolic Surgery, King’s College Hospital, London, SE5 9RS UK; 7 School of Sport, Exercise and Applied Science, St Mary’s University, Twickenham, London, TW1 4SX UK; 8 General and Digestive Surgery Department of Hospital Del Mar de, 08003 Barcelona, Spain; 9 Faculty of Medicine, University of São Paulo, São Paulo, SP 05508-220 Brazil; 10 Division of Medicine, University College London, London, WC1E 6JF UK

**Keywords:** Bariatric surgery, Metabolic surgery, Roux-en-Y gastric bypass, Sleeve gastrectomy, Nutritional intake, Dietary intake, Micronutrient, Macronutrient

## Abstract

The relationship between postoperative dietary intake and weight loss after bariatric surgery remains unclear. We performed a systematic review and meta-analysis of studies published between January 2000 and May 2023, reporting weight loss outcomes, and dietary intake before and after Roux-en-Y gastric bypass and sleeve gastrectomy. A total of 42 studies were included. There was no detectable difference in dietary intake between the two procedures. Roux-en-Y gastric bypass induced an average decrease in energy intake of 886 kcal/day at 12-month post-surgery; however, there was no correlation between daily energy intake and weight loss. These findings show a substantial reduction of energy intake in the first year after bariatric surgery but do not support a link between lower energy intake and greater weight loss.

**Graphical Abstract:**

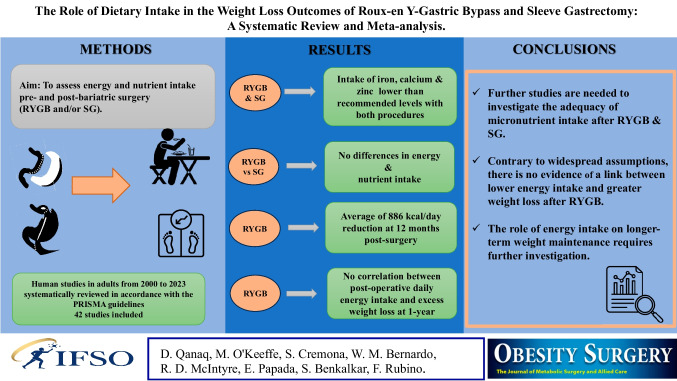

## Introduction

Bariatric surgery is the most effective treatment for severe obesity [1–3]. Roux-en-Y gastric bypass (RYGB) and sleeve gastrectomy (SG) are the most commonly performed procedures [4]. Both types of these bariatric procedures exert considerable effects on energy and nutrient intake, especially in the first 6–12 months, owing to their ability to influence mechanical and physiological mechanisms involved in the regulation of hunger and satiety [5]. However, the relative role of changes in energy and nutrient intake in determining weight loss and particularly long-term weight loss maintenance remains unclear.

A systematic review by Janmohammadi et al. (2019) evaluated the impact of bariatric surgery on energy and nutrient intake, specifically total energy and macronutrients. They reported that bariatric surgery significantly reduced total energy intake and increased fat and protein intake but observed no effect on carbohydrates. These findings offer some understanding of the impact of surgery on energy and macronutrient intake; however, the conclusions were based on observations made over a very broad range of time, from 3 months to 8 years postoperatively. Therefore, it is not possible to discriminate between changes that occur at short- and long-term time points after surgery and their relative role in the mechanisms of surgical weight loss. Additionally, postoperative macronutrient intakes that fall within the acceptable macronutrient distribution ranges (AMDRs; 45% carbohydrate, 20% fat, 35% protein, as percent of total energy) have been shown to optimize surgical outcomes and reduce complications [6–9], yet no study to date has compared postoperative intakes to the AMDRs. The impact of bariatric surgery on micronutrient intake in the short- and long-term has also not been systematically evaluated, despite the known risk of micronutrient deficiency after bariatric surgery [10, 11].

Understanding whether changes in energy and nutrient intake that occur early after surgery persist long-term and whether anatomically distinct procedures differentially affect nutrient intake has both clinical and mechanistic interest. This knowledge could help optimize surgical outcomes and identify elusive mechanisms of gastrointestinal (GI) physiology that could serve as a target for novel anti-obesity interventions. This systematic review and meta-analysis investigated the impact of bariatric surgery, specifically RYGB and SG, on nutrient intake in the short and long term and evaluated intakes against the recommended AMDRs. A specific objective of this review was also to assess evidence of a role of daily energy intake as a major determinant of weight loss after bariatric surgery.

## Methods

This systematic review has been conducted in accordance with the PRISMA guidelines for reporting systematic reviews. Studies reporting on the effects of RYGB or SG on energy intake and/or macro/micronutrient intake were included.

### Database Searches

Embase, Clinical Trials, Google Scholar, and PubMed databases were searched by two independent reviewers (DQ, B.WM). The search included all articles published between January 2000 and May 2023. The search strategy included medical subject headings (MeSH) terms and the following key search terms: ((((Roux-en-Y Gastric Bypass) OR (Bypass, Gastric) OR (Bypass, Roux-en-Y Gastric) OR (RYGB), OR (Sleeve gastrectomy) OR (SG) (Bariatric surgery) OR (weight-loss surgery) OR (metabolic surgery)) AND ((diet) OR (dietary intake) OR (diet intake) OR (nutrition) OR (nutrient) OR (nutritional intake) OR (food intake) OR (energy intake)) NOT ((gastric band surgery) OR gastric banding)))). Filters applied were full text, humans, adult: 19 + years, English, multi- and single-center study, clinical study, clinical trial, observational study, comparative studies, controlled clinical trial, and randomized control trial. The identified literature was stored in Mendeley reference manager.

### Inclusion and Exclusion Criteria

Inclusion criteria included participants > 18 years of age undergoing RYGB or SG, outcome data that reported on dietary or nutrient intake, including total energy, macro- and micro-nutrient intake. Only studies published between January 2000 and May 2023 were included. This period was chosen to reflect the changes in the contemporary food environment, industries, products, and surgical procedures. All observational study designs were included. Non-English language articles (*n* = 64) were excluded during the initial search. The decision to exclude these articles was based on considerations of language proficiency and resource constraints. Animal studies, studies investigating other bariatric procedures, studies limited to the pre-operative period, and those assessing taste preferences and/or nutritional status (biomarkers) but not dietary or nutrient intake were excluded. Interventional studies that altered the dietary intake of the participants or supplemented protein, lipid, or carbohydrates were excluded. Review articles, meta-analyses, and editorials were also excluded.

### Data Extraction

**Fig. 1:**
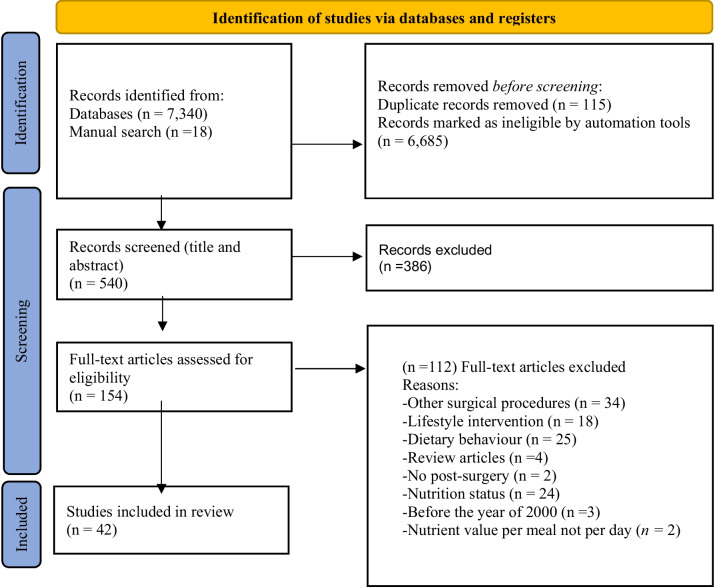
PRISMA 2020 flow diagram for systematic reviews which included searches of databases and registers only. From: Page MJ, McKenzie JE, Bossuyt PM, Boutron I, Hoffmann TC, Mulrow CD, et al. The PRISMA 2020 statement: an updated guideline for reporting systematic reviews. BMJ 2021;372: n71. https://doi.org/10.1136/bmj.n71

Following the removal of duplicates, titles, and abstracts were screened in triplicate against the inclusion and exclusion criteria; reasons for exclusion are outlined in Fig. 1. Data extraction from full-text articles was undertaken independently by two reviewers and included study characteristics and the pre-specified outcomes. Hand-searching of reference lists of eligible papers was also undertaken. The extracted data were synthesized using qualitative techniques, including a narrative summary approach to facilitate the interpretation of results. Short-term follow-up was considered as ≤ 12 months while studies reporting data > 12 months were classified as long term (Table 1).

**Table 1:** Study characteristics of studies included in the systematic review

Author	Year	Country	Study design	Surgery type	Sample size	% female	Age, yeas	F/U time-points (months)
Molin Netto et al.	2016	Brazil	Prospective—L	RYGB	41	95	39 ± 11	0, 6
Sarwer et al.	2008	USA	Prospective—L	RYGB	200	82	43 ± 10	0, 4, 9, 15, 21
Gesquiere et al.	2017	Belgium	Prospective—L	RYGB	54	61	48(47–49)*	0, 1, 3, 6, 12
Ruz et al.	2009	Chile	Prospective—L	RYGB	67	100	37 ± 10	0, 1, 3, 6, 12, 18
Miller et al.	2014	USA	Prospective—L	RYGB	17	94	47 ± 2	0, 1, 3, 6, 12
Bobbioni-Harsch et al.	2002	Switzerland	Prospective—L	RYGB	50	100	38 ± 1	0, 3, 6, 12
Da Silva et al.	2014	Brazil	Prospective—L	RYGB	10	100	47 ± 7	0, 3
Moize et al.	2003	Spain	Prospective—L	RYGB	93	83	41 ± 12	3, 6, 12
Ferreira Nicoletti et al.	2013	Brazil	Prospective—L	RYGB	30	100	36 ± 9	0, 3, 6, 12
Mercachita et al.	2014	Portugal	Prospective—L	RYGB	60	65	42 ± 12	0, 12, 24
Dias et al.	2006	Brazil	Prospective—L	RYGB	40	100	43 ± 11	3,6,9,12
Laurenius et al.	2013	Sweden	Prospective—L	RYGB	43	72	43 ± 10	0,1,12,24
Bavaresco et al.	2010	Brazil	Prospective—L	RYGB	48	85	48 ± 9	0,1,3,6,8,12
Carrasco et al.	2012	Chile	Prospective—L	RYGB	50	100	38 ± 10	0, 6, 12
Pinto et al.	2019	Brazil	Prospective—L	RYGB	58	68	39 ± 9	0, 3, 12
Kruseman et al.	2010	Switzerland	Prospective—L	RYGB	80	100	40 ± 10	0, 96
Giusti et al.	2016	Switzerland	Prospective—L	RYGB	16	100	39 ± 2	0, 3, 6, 12, 36
Leite Faria et al.	2009	Brazil	CS	RYGB	75	80	37 ± 11	23 ± 10
Reid et al.	2016	Canada	CS	RYGB	27	88	3 ± 8	12 ± 3.7
Ortega et al.	2012	Spain	CS	RYGB	107	79	42 ± 10	36
Silveira et al.	2014	Brazil	CS	RYGB	36	100	43 ± 10	20
Freire et al.	2011	Brazil	CS	RYGB	100	84	45 ± 10	46 ± 33
Benson-Davies et al.	2013	USA	CS	RYGB	24	100	52 ± 11	72
Lopes Da Silva et al.	2016	Brazil	CS	RYGB	80	88	46 ± 16	47 ± 18
Wardé-Kamar et al.	2004	USA	CS	RYGB	69	92	46 ± 11	48 ± 11
Ortega et al.	2016	Spain	CS	RYGB	107	79	42 ± 10	0, 36
Goode et al.	2004	USA	CS	RYGB	26	100	48 ± 9	36
De Torres Rossi et al.	2012	Brazil	Retrospective- CC	RYGB	82	82	45 ± 10	41
Schieferdecker et al.	2018	Brazil	Retrospective- L	RYGB	106	91	48	0, 3, 6, 12
Taylor et al.	2019	Poland	Prospective- L	SG	154	53	48 (40–54)^*^	0, 3, 6, 12
Dagan. et al.	2017	Israel	Prospective- L	SG	77	57	43 ± 9	0, 1, 3, 6, 12
Gjessing et al.	2013	Norway	Prospective- L	SG	150	77	44 (34–51)^*^	0, 3, 12
Coluzzi et al.	2016	Finland	Prospective- L	SG	30	73	35	0, 1, 3, 6, 12, 24
Chou et al.	2017	Taiwan	Retrospective- CC	SG	40	50	34	60
Golzarand et al.	2018	Iran	Prospective- L	RYGB + SG	43	100	41 ± 7	0,6
Verger et al.	2015	French	Prospective- L	RYGB + SG	52	68	43 (38–51)^*^	0, 3, 6, 12
Moizé et al.	2013	Spain	Prospective- L	RYGB + SG	50	82	44 ± 2	0, 1, 4, 8, 12
Coupaye et al.	2013	French	Prospective- L	RYGB + SG	43	72	44 ± 9 RYGB	0, 6, 12
							45 ± 11 SG	
Moizé et al.	2013	Spain	Prospective- L	RYGB + SG	355	75	45 ± 11 RYGB	0, 6, 12, 24, 48, 60
							46 ± 12 SG	
Lim et al.	2020	Korea	Prospective- L	RYGB + SG	189	71	35 ± 11	0, 1, 6, 12
El Labban et al.	2015	Lebanon	CS	RYGB + SG	60	60	40 ± 11	0, 6
Barstad et al.	2023	Norway	CS	RYGB + SG	109	66	48 ± 10	0, 12

Abbreviations: *CC* case control, *CS* cross-sectional, *L* longitudinal, *RYGB* Roux-en-Y gastric bypass, *SG* sleeve gastrectomy

Data are expressed as mean ± SD unless otherwise indicated

^*^Data are expressed as median (IQR)

### Key Dietary Measures

The dietary outcomes included total energy intake (kcal/day), macronutrient intake as a percentage of total energy or grams, micronutrient composition as a percentage of energy or amount (mg or µg/day), or amount of the nutrient as a proportion of dietary reference intake (DRI).

### Methods of Meta-analysis

A meta-analysis was performed to investigate the impact of RYGB on total energy intake and macronutrients (%En). The meta-analysis was completed using the “metacont” command from the meta package of R software, version 4.2.3 [12].

Additionally, the meta-analysis was conducted using the “metamean” command from the “meta” package in R software, version 4.3.1 [12], and it assessed total energy intake (kcal/day), macronutrients composition as a percentage of energy (%En), before bariatric surgery and 6- and 12-month post-surgery. Quantitative variables were expressed as mean ± standard deviation (SD). The random-effects model was applied because observational studies are inherently heterogeneous. The statistical method used to weight the measures of association among the included studies was the inverse of the variance [raw means (MRAW)].

The primary criterion for including studies in the meta-analysis was the presence of complete data (mean and SD) at the specified time points (0, 6, 12, and 24 months). The second criterion was to refrain from analyzing two different types of bariatric surgery (RYGB and SG) together. Since there are limited studies at the 24-month time point, RYGB at the specified time points including 0, 6, and 12 months were chosen for the analysis (*n* = 16).

### Energy Intake and Weight Loss

A secondary objective of this review was to determine if daily energy intake is a major determinant of weight loss. Hence, we conducted a meta-analysis to investigate the relationship between average daily energy intake and excess weight loss (EWL) at 12-month post-surgery. To classify the results and establish a comparison parameter, a modification of the Reinhold classification was applied. In this modified classification, an excellent outcome is defined as a reduction in excess weight greater than 75%, a good result falls within a range of 50 to 75% reduction in excess weight, and a failure occurs if the weight loss is less than 50%.

## Results

The initial search identified 7322 articles. Following the removal of duplicates and studies that did not meet the eligibility criteria, 540 studies were screened for relevance by title and abstract review, and an additional 18 records were identified through a manual search of the reference lists of eligible articles. Further details are presented in the PRISMA diagram (Fig. 1). A total of 42 studies were eligible for full-text data extraction and analysis.

### Study Design

Of the 42 studies included, 27 were longitudinal prospective studies, 13 were cross-sectional, and two were case–control studies.

### Demographic and Clinical Characteristics

The age of participants ranged between 30 and 50 years. Participants were predominantly female; specifically, 59% (*n* = 24) of the studies had more females than males, 29% (*n* = 12) had female-only participants, and 12% (*n* = 5) had a similar number of males and females. The mean sample size across studies was 74 ± 63 participants and ranged from 10 [13] to 355 [14] participants. The follow-up period ranged from 6 months [15–17] to 8 years of post-surgery [18]. Twenty-nine (70%) studies utilized RYGB, five (12%) SG and seven (17%) studies compared RYGB and SG**.**

Twenty-four percent of studies (*n* = 10) were conducted between 2000 and 2010, 42% (*n* = 17) between 2011 and 2015, and 34% (*n* = 14) were between 2016 and 2020. There were 15% (*n* = 6) from North America, 39% (*n* = 16) from Europe, 34% (*n* = 14) from South America, 7% (*n* = 3) from the Middle East, and 5% (*n* = 2) from East Asia.

### Methods of Dietary Assessment

 Various dietary assessment methods were employed across different studies. Nineteen (46%) studies utilized a food recall [13, 16, 18–30], and seventeen (41%) used dietary records [14, 28, 31–40], while the remaining studies used food frequency questionnaires (*n* = 8; 20%) [17, 41–45] or employed mixed methods (*n* = 5; 12%) [15, 17, 41, 45, 46]. The best method regarding flexibility, accuracy, and reflection of the actual diet is a dietary diary. Food diaries do not rely on memory and thus are considered more accurate than 24-h food recall and food frequency questionnaires (FFQs) [47]. In addition, FFQs are restricted to specific food items of the questionnaire and may fail to fully capture habitual dietary intake. Dietary assessment is crucial to accurately assess the impact of bariatric surgery on dietary intake and the impact of dietary intake on surgical outcomes.

Daily energy (kcals) was reported in 90% of studies (*n* = 37) [8, 13–46, 48–52]; all studies reported macronutrient intake whereas only ten studies (24%) [14, 19, 21, 26, 30, 33, 35, 38, 43, 45] reported micronutrient intake.

### Studies Comparing RYGB and SG

Only seven (17%) studies investigated the difference in energy and nutrient intake between RYGB and SG [8, 14–16, 24, 25, 48]. Six of these comparative studies were conducted over a short period of postoperative observation (6–12-month post-surgery) and found no significant differences in dietary intake, including energy and macronutrient intake, between RYGB and SG [8, 15, 16, 24, 25, 48]. Additionally, no differences in weight loss between RYGB and SG were reported. Only one of the seven studies investigated the long-term (> 12-month post-surgery) difference in dietary intake [14]. This was a 5-year prospective study, and its results also show no difference in dietary intake or weight loss outcomes.

### Energy and Macronutrient Intakes

Energy and macronutrient intakes were assessed using a dietary diary in 54% of the included studies, whereas 15% used FFQs and 39% used 24-h dietary recalls.

The weighted mean (WM) of total energy intake before surgery was 2049 kcal/day (95% CI: 1845; 2252). There was a decrease in energy intake at 6-month post-surgery; WM, 1038 kcal/day (95% CI, 935; 1141), followed by a slight increase at 12-month post-surgery WM, 1284 kcal/day (95% CI, 1134; 1433) [8, 18, 27–29, 38–40, 42–46, 52, 53].

Carbohydrate intakes remained relatively unchanged by surgery; WM of intake before surgery was 49%En (95% CI: 43; 54), 44%En (95% CI: 40; 49) at 6 months and 46%En (95% CI: 42; 49) at 12-month post-surgery [19, 21, 22, 24–26, 31–37, 40, 48, 49, 51, 52, 54]. Carbohydrate intake pre-surgery and at all time points, post-surgery was within the AMDRs (45–65%En).

Protein intake before surgery had a WM of 16%En (95% CI: 13; 19), and intake increased to 19%En (95% CI, 16; 22) at 6 months, which was maintained at 12 months 18%En (95% CI, 16; 21) [8, 18, 27–29, 38–40, 42–46, 52, 53]. Protein intake pre-surgery and at all time points, post-surgery was within the AMDRs (10–35%).

Fat intake WM was 36%En (95% CI: 32; 41) before surgery and remained unchanged at 6- and 12-month post-surgery (6 months 36%En (95% CI: 31; 41); 12 months 36% En (95% CI: 33; 39)) [8, 18, 27–29, 38–40, 42–46, 52, 53]. Fat intake was slightly higher than AMDRs (25–35%) pre-surgery and at all time points after surgery.

**Fig. 2:**
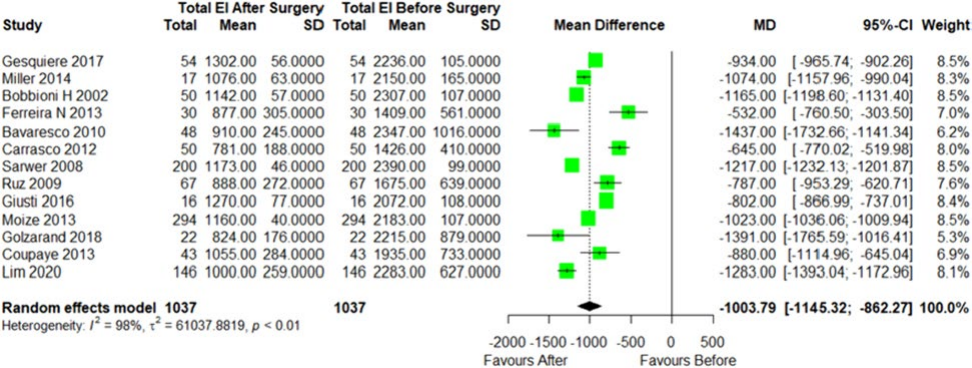
Forest plot of energy intake before and 6 months after RYGB. SD, standard deviation; CI, confidence interval

**Fig. 3:**
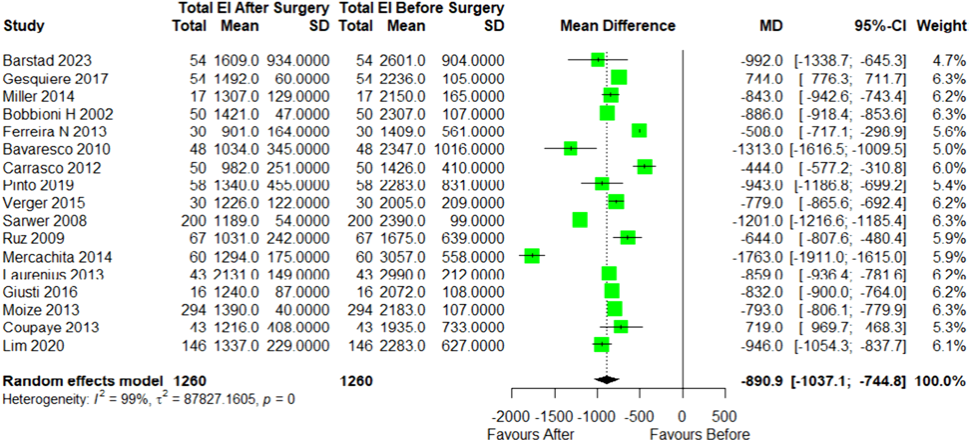
Forest plot of energy intake before and 12 months after RYGB. SD, standard deviation; CI, confidence interval

The change in dietary intake following RYGB surgery in short term (< 12 months) was more commonly reported than long term (> 12 months), therefore the meta-analysis was only performed on short-term data, < 12 months. There was a significant reduction in energy intake by 1003 kcal/d (MD =  − 1003, 95% CI − 1145 to − 862; *p* < 0.001) at 6 months compared to before RYGB (Fig. 2). A significant reduction in energy intake from baseline was also observed at 12-month post-RYGB by a mean of 886 kcal/d (MD =  − 886, 95% CI − 1039 to − 732; *p* < 0.001) (Fig. 3).

Compared to pre-surgery intakes there was no significant difference in any of the macronutrients (%En) at 6 months (carbohydrates MD =  − 4.3, 95% CI − 9.0 to 0.5, *p* < 0.08; protein MD = 3.4, 95% CI − 2.0 to 8.8, *p* = 0.22; fat MD =  − 0.23, 95% CI − 1.8 to 1.4; *p* = 0.77) or 12 months after RYGB (carbohydrates MD =  − 2.7, 95% CI − 6.5 to 1.1, *p* = 0.17; protein MD = 1.8, 95% CI − 2.1 to 5.7, *p* = 0.36); fat MD =  − 0.01, 95% CI − 1.6953 to 1.6731, *p* = 0.99) Table 2.

### Micronutrients Intake

Only ten studies investigated the micronutrient intake following RYGB and/or SG [14, 19, 21, 26, 30, 33, 35, 38, 43, 45]. As summarized in Table 2, in the short term (≤ 12 months), vitamin B12 intakes met the UK RDA of 2.4 µg/day in all the studies while zinc intake was < 8 mg/day in two studies [33, 35] at 3- and 12- month post-surgery. Dias et al. reported iron intake < 8 mg/day at 3- and 12-month post-surgery [33]. In the long term (> 12-month post-surgery), Silveria et al. reported vitamin B12 and zinc intakes in line with the UK RDAs, but iron remained suboptimal at 20-month post-surgery (Table 3; [43]). Overall, both surgical procedures resulted in lower intakes of folic acid, vitamins D, E, C, and calcium, in the long term [14, 26, 30, 38, 39, 45].

**Table 2:** Studies reporting short-term (0 to 12 months) changes in post-operative weight loss, energy, and nutrient intake

Author, year	Dietary assessment	F/U, months	Weight loss, %EWL or %WL*	Total EI, kcal/d	CHO, %En	Protein, %En	Fat, %En	Vit B12, mcg/d	Zinc, mg/d	Iron, mg/d
Molin Netto et al. 2016	FFQ	0	-	2900	50	20	28	-	-	-
		6	EWL 63 ± 25	1000	54	21	23	-	-	-
Gesquiere et al. 2017	2-day FR	0	-	2236 (2053–2418)*	-	-	-	6(5–6)*	57(32–82)*	13(12–13)*
		3		1085 (987–1183)*	-	-	-	4(3–4)*	11(8–13)*	12(5–17)*
		6	EWL 58 (55–61)*	1302 (1205–1399)*	-	-	-	4(4–5)*	11(9–14)*	23(11–34)*
		12	EWL 71 (68–73)*	1492 (1387–1596)*	-	-	-	5(4–5)*	17(15–18)*	42(29–56)*
Leite Faria et al. 2009	4-day FR	0	-	-	-	-	-	-	-	-
		3	EWL 68 ± 19	-	-	-	-	-	-	-
Miller et al. 2014	4-day FR	0	-	2150 ± 165	45 ± 2	7 ± 1	39 ± 2	6 ± 1	12 ± 1	18 ± 2
		3	-	877 ± 60	36 ± 1	28 ± 2	36 ± 2	3 ± 1	6 ± 1	8 ± 1
		6	EWL 46 ± 3	1076 ± 63	37 ± 2	27 ± 1	38 ± 2	4 ± 1	8 ± 1	8 ± 1
		12	EWL 58 ± 4	1307 ± 129	42 ± 2	23 ± 1	37 ± 2	5 ± 1	8 ± 1	10 ± 1
Bobbioni-Harsch et al. 2002	24-h DR +	0	-	2307 ± 107	43 ± 1	15 ± 0	41 ± 1	-	-	-
	3-day FR	3	EWL 40 ± 2	966 ± 42	39 ± 1	18 ± 1	42 ± 1	-	-	-
		6	EWL 58 ± 2	1142 ± 57	39 ± 1	17 ± 1	43 ± 1	-	-	-
		12	EWL 75 ± 3	1421 ± 47	42 ± 1	16 ± 1	42 ± 1	-	-	-
Lopes Da Silva et al. 2016	24-h DR × 2	0	-	1519 ± 291	52 ± 9	17 ± 3	33 ± 9	-	-	-
		3	-	995 ± 276	48 ± 13	20 ± 7	34 ± 10	-	-	-
Moize et al. 2003	24-h DR	0	-	-	-	-	-	-	-	-
		3	EWL 27 ± 9	772 ± 323	47 ± 11	24 ± 8	28 ± 7	-	-	-
		6	EWL 39 ± 13	866 ± 320	45 ± 15	25 ± 10	30 ± 11	-	-	-
		12	EWL 49 ± 17	1101 ± 400	47 ± 11	23 ± 6	30 ± 1	-	-	-
Ferreira Nicoletti et al. 2013	24-h DR	0	-	1409 ± 561	55 ± 24	21 ± 6	25 ± 1	-	-	-
		3	-	798 ± 286	51 ± 27	22 ± 9	28 ± 11	-	-	-
		6	-	877 ± 305	52 ± 27	22 ± 7	26 ± 10	-	-	-
		12	EWL 63 ± 9	901 ± 164	51 ± 14	21 ± 8	28 ± 8	-	-	-
Dias et al. 2006	24-h DR	0	-	-	-	-	-		-	-
		3		529 ± 47	-	-	-	4 ± 2	4 ± 1	5 ± 1
		6	-	1369 ± 262	-	-	-	-	-	-
		12	EWL 67	866 ± 95	-	-	-	3 ± 1	4 ± 1	6 ± 1
Bavaresco et al. 2010	24-h DR	0	-	2347 ± 1016	47 ± 20	20 ± 9	33 ± 4	-	-	-
		3	-	796 ± 306	55 ± 19	19 ± 10	28 ± 16	-	-	-
		6	-	910 ± 245	53 ± 18	19 ± 8	30 ± 11	-	-	-
		12	-	1034 ± 345	53 ± 21	18 ± 8	31 ± 12	-	-	-
Carrasco et al. 2012	3-day FR	0	-	1426 ± 410	53 ± 19	17 ± 4	30 ± 13	-	-	-
		6	EWL 59 ± 11	781 ± 188	49 ± 13	24 ± 8	29 ± 10	-	-	-
		12	EWL 69 ± 13	982 ± 251	53 ± 16	21 ± 9	32 ± 9	-	-	-
Schieferdecker et al. 2018	24-h DR	0	-	-	-	-	-	-	-	-
		3	EWL 54 (24–113)	-	35	-	26	-	7	-
		6	EWL 73 (33–139)	-	40	-	32	-	5	-
		12	EWL 88 (36–150)	-	50	-	40	-	5	-
Pinto et al. 2019	3 × 24-h DR	0	-	2283 ± 831	48 ± 9	18 ± 4	33 ± 6	-	-	-
		3	EWL 47 ± 11	1038 ± 2767	47 ± 7	25 ± 6	27 ± 6	-	-	-
		12	EWL 84 ± 19	1340 ± 455	48 ± 9	21 ± 4	31 ± 6	-	-	-
Da Silva et al. 2014	7-day FR	0	-	1519 ± 292	53 ± 9	17 ± 3	33 ± 8	-	-	-
		3	-	995 ± 276	48 ± 13	20 ± 7	34 ± 10	-	-	-
Dagan et al. 2017	3-day FR	0		2145 ± 939	-	-	-	-	-	-
		3	EWL 45 ± 11	841 ± 355	-	-	-	-	-	-
		6	EWL 63 ± 13	1109 ± 458	-	-	-	-	-	-
		12	EWL 77 ± 18	1317 ± 493	-	-	-	-	-	-
Hanne et al. 2013	24-h DR	0			-	-	-	-	-	-
		3	EWL 46 (38–56)*	710 (474–881)*	-	-	-	-	-	-
		12	EWL 72 (59–87)*	918 (728–1105)*	-	-	-	-	-	-
Golzarand et al. 2018	3-day FR	0		2215 ± 879 RYGB	52 ± 10 RYGB	14 ± 3RYGB	34 ± 8 RYGB	-	-	-
		6	EWL 52 ± 13 RYGB	824 ± 176 RYGB	47 ± 14 RYGB	18 ± 7 RYGB	35 ± 9 RYGB	-	-	-
		0		2565 ± 1065 SG	46 ± 9 SG	17 ± 4 SG	38 ± 9 SG	-	-	-
		6	EWL 66 ± 24 SG	844 ± 256 SG	46 ± 7 SG	17 ± 4 SG	37 ± 10 SG	-	-	-
Verger et al. 2015	3-day FR	0		2005 (1539–2266)* RYGB	48 (42–50)* RYGB	17(16–19)* RYGB	32(30–41)* RYGB	-	-	-
		3	WL 23 (20–27)* RYGB	711 (615–1006)* RYGB	44 (39–49)* RYGB	24(16–20)* RYGB	37(32–39)* RYGB	-	-	-
		6	WL 32 (28–38)* RYGB	-	-	-	-	-	-	-
		12	WL 39 (29–49)* RYGB	1226 (1133–1559)* RYGB	42 (35–47)* RYGB	16(13–17)* RYGB	38(33–45)* RYGB	-	-	-
		0		1658 (1445–2395)* SG	44(40–47)* SG	19(17–20)* SG	37(33–40)* SG	-	-	-
		3	WL 18 (15–23)* SG	833 (539–1108)* SG	37(32–47)* SG	20(19–20)* SG	42(36–45)* SG	-	-	-
		6	WL 24 (19–30)* SG	-	-	-	-	-	-	-
		12	WL 27 (26–33)* SG	1078 (793–1354)* SG	42(33–45)* SG	19(18–20)* SG	39(37–44)* SG	-	-	-
Moizé, et al. 2013	24-h DR	0	-	2246 ± 108	37 ± 1	17 ± 1	45 ± 1	-	-	-
		4	-	886 ± 31	37 ± 1	26 ± 1	37 ± 1	-	-	-
		12	-	1253 ± 56	38 ± 1	21 ± 1	41 ± 1	-	-	-
Coupaye et al. 2013	4-day FR +	0	-	1935 ± 733 RYGB	46 RYGB	18 RYGB	36 RYGB	-	-	-
	interview	6	WL 32 ± 9 RYGB	1055 ± 284 RYGB	43 RYGB	20 RYGB	37 RYGB	-	-	-
		12	WL 39 ± 10 RYGB	1216 ± 408 RYGB	48 RYGB	18 RYGB	34 RYGB	-	-	-
		0	-	1712 ± 590 SG	46 SG	19 SG	35 SG	-	-	-
		6	WL 29 ± 14 SG	1045 ± 336 SG	44 SG	19 SG	37 SG	-	-	-
		12	WL 36 ± 18 SG	1203 ± 349 SG	43 SG	19 SG	37 SG	-	-	-
Lim et al. 2020	3-day FR	0	Success‡	2283 ± 627	54	17	30		-	
		6	Success‡	1000 ± 259	41	25	34	-	-	-
		12	Success‡	1337 ± 229	48	28	24	-	-	-
		0	Failure‡	2234 ± 609	56	15	29	-	-	-
		6	Failure‡	1121 ± 272	46	20	33	-	-	-
		12	Failure‡	1646 ± 316	53	20	27	-	-	-
L.H. Barstad et al. 2023	FFQ	0	-	2801 (2552–3049)* SG	42(40–43)* SG	18(17–19)* SG	37(36–39)* SG	10(9–12)* SG	18(15–19)* SG	16(7–25)* SG
		12	-	1462 (1207–1716)* SG	40(42–39)* SG	20(19–20)* SG	36(35–38)* SG	10(9–11)* SG	26(24–28)* SG	36(26–45)* SG
		0	-	2601 (2354–2850)* RYGB	42(40–44)* RYGB	18(17–18) *RYGB	37(36–39)* RYGB	9(7–10)* RYGB	16(14–18)* RYGB	15(6–24)* RYGB
		12	-	1609 (1354–1863)* RYGB	42(43–40)* RYGB	19(18–20)* RYGB	35 (34–37)* RYGB	11(10–12)* RYGB	30(28–32)* RYGB	54(44–63)* RYGB

Abbreviations: *DR* dietary record, + more than one dietary method, *EI* energy intake, *FFQ* food frequency questionnaire, *FR* food record, *EWL* excess weight loss, *RYBG* Roux-en-Y gastric bypass, *SG* sleeve gastrectomy, *WL* weight loss

Values are expressed as mean ± SD unless otherwise indicated

^*^Data expressed as median (IQR)

^‡^Success and failure groups depend on whether participants achieved 50% loss of excess weight at 12 months, please refer to the original study for further information

**Table 3:** Studies reporting post-operative long-term (>12 months) changes in weight loss, energy, and nutrient intake

Author, year	Dietary assessment	F/U, months	Weight loss, %EWL or %WL*	Total EI, kcal/d	CHO, %En	Protein, %En	Fat, %En		Vit B12, mcg/d	Zinc, mg/d	Iron, mg/d
Sarwer et al. 2008	FFQ	0	-	2390 ± 99	44 ± 1	15 ± 0		41 ± 1	-	-	-
		6	WL 35 (34–36)*	1173 ± 46	45 ± 1	16 ± 0		40 ± 1	-	-	-
		12	WL 39 (38–41)*	1189 ± 54	45 ± 1	16 ± 1		40 ± 1	-	-	-
		16	WL 39 (37–41)*	-	43 ± 1	16 ± 2		42 ± 1	-	-	-
		24	-	-	-	-		-	-	-	-
Ruz et al. 2009	3-day FR	0	-	1675 ± 639	-	16 ± 5		-	-	-	9 ± 5
		6	-	888 ± 272	-	10 ± 4		-	-	-	6 ± 3
		12	-	1031 ± 242	-	12 ± 4		-	-	-	7 ± 2
		18	-	1178 ± 390	-	13 ± 4		-	-	-	8 ± 4
Mercachita et al. 2014	24-h DR	0	-	3057 ± 558	-	-		-	4 ± 1	-	12 ± 3
		12	EWL 70 ± 15	1294 ± 175	-	-		-	2 ± 0	-	6 ± 2
		24	EWL 63 ± 10	1520 ± 200	-	-		-	2 ± 1	-	7 ± 2
Laurenius et al. 2013	FFQ	0	-	2990 (2619–3354)*	-	-		37 (34–39)*	-	-	-
		12	WL 31 ± 7	2131 (1873–2390)*	-	-		34 (33–36)*	-	-	-
		24	WL 32 ± 9	2425 (2103–2591)*	-	-		35 (33–36)*	-	-	-
Ortega et al. 2012	72-h DR	0	-	-	-	-		-	-	-	-
		36 (12–84)*	EWL 61 ± 23	1364 ± 293	47	15		38	-	-	-
Silveira et al. 2014	FFQ	0	-	-	-	-		-	-	-	-
		20	-	-	-	-		-	2.5	-	5.5
Reid et al. 2016	3-day FR	145 ± 44	WL 35 ± 14	1705 ± 573	43 ± 11	19 ± 6		35 ± 11	-	-	-
Ortega et al. 2016	3-day FR	36 ± 24	EWL 77 ± 24	1364 ± 293	47	15		38	-	-	-
Goode et al. 2004	FFQ	≥ 36	-	1582 ± 54	54 ± 23	17 ± 1		30 ± 5	-	-	-
Kruseman et al. 2020	4-day FR	0	-	2271 ± 769	41 ± 7			42 ± 6	-	-	-
		96	EWL 56 ± 23	1680 ± 506	42 ± 9			40 ± 8	-	-	-
Giusti et al. 2016	7-day FR	0	-	2072 ± 108	62 ± 2	17 ± 1		39 ± 3	-	-	-
		3	-	1047 ± 62	42 ± 3	17 ± 1		40 ± 3	-	-	-
		6	-	1270 ± 77	43 ± 4	16 ± 1		43 ± 5	-	-	-
		12	WL 34 ± 1	1240 ± 87	45 ± 3	15 ± 1		40 ± 4	-	-	-
		36	-	1448 ± 57	40 ± 2	16 ± 1		47 ± 3	-	-	-
Benson-Davies et al. 2013	7-day FR	72	-	1429 ± 411	43	17		39	-	-	-
Wardé-Kamar et al. 2004	24-h DR	18–48	EWL 58 ± 17	2012 ± 985	45 ± 11	22 ± 7		32 ± 11	-	-	-
Freire et al. 2011	24-h DR +	24	-	949 ± 334	-	-		-	-	-	-
	FFQ	24–60	-	1343 ± 530	-	-		-	-	-	-
		> 60	-	1169 ± 426	-	-		-	-	-	-
		Total population	EWL 59 ± 20	1152 ± 462	51 ± 9	15 ± 4		34 ± 8	-	-	-
De Torres Rossi et al. 2012	4-day FR	> 12 (41)*	EWL 78	1383 ± 474	39 (38–41)*	17 (16–18)*		43 (42–44)*	2 (1–22)*	-	-
Coluzzi et al. 2016	24-h DR	0		3002 (1824–5533)*	47	17		36	-	-	-
		3	EWL 38 (11 65)*	891 (682–1100)*	25	19		57	-	-	-
		6	EWL 60 (22–83)*	961 (786–1193)*	44	23		33	-	-	-
		12	EWL 65 (34–94)*	1006 (938–1133)*	42	22		36	-	-	-
		24	EWL 72 (40–101)*	900 (693–1244)*	42	27		31	-	-	-
Chou et al. 2017	FFQ	0		-	-	-		-	-	-	-
		12	EWL 105 ± 33	-	-	-		-	-	-	-
		60	EWL 96 ± 33	1230	41	23		36	-	8	8
El Labban et al. 2015	FFQ +	22 RYGB	EWL 70 RYGB	1555 ± 657	41	14		37	-	-	-
	3 × 24 h DR		EWL 81 SG	1373 ± 606	42	14		37			
Moizé et al., 2013	3-day FR +	0	-	2187 (2210–2254) *RYGB	40 (39–41)* RYGB	18 (18–19)* RYGB		42 (40–43)* RYGB	-	-	14 (12–15)* RYGB
	24 h DR	6	-	1160 (1089–12311)* RYGB	40 (39–42)* RYGB	21 (21–22)* RYGB		39 (37–40)* RYGB	-	-	9 (7–10)* RYGB
		12	-	1390 (1320–1460)* RYGB	40 (39–41)* RYGB	20 (19–20)* RYGB		40 (39–42)* RYGB	-	-	9 (8–11)* RYGB
		24	-	1533 (1455–1611)* RYGB	40 (38–41)* RYGB	19 (18–20)* RYGB		41 (40–43)* RYGB	-	-	10 (8–11)* RYGB
		48	-	1581 (1483–1680)* RYGB	40 (39–42)* RYGB	18 (17–19)* RYGB		42 (40–44)* RYGB	-	-	10 (8–13)* RYGB
		60	-	1561 (1473–1690)* RYGB	41 (39–43)* RYGB	19 (18–20)* RYGB		40 (38–42)* RYGB	-	-	9 (6–11)* RYGB
		0	-	2283 (2136–2430)* SG	40 (37–43) *SG	18 (17–20)* SG		42 (39–45)* SG	-	-	14 (12–18)* SG
		6	-	1163 (1013–1314)* SG	38 (36–41)* SG	23 (22–25)* SG		39 (36–41)* SG	-	-	8 (5–12)* SG
		12	-	1319 (1162–1475)* SG	35 (32–38)* SG	22 (20–23)* SG		42 (39–45)* SG	-	-	17 (13–20)* SG
		24	-	1469 (1288–1650)* SG	43 (39–46)* SG	20 (19–22)* SG		38 (35–42)* SG	-	-	9 (5–13)* SG
		48	-	1409 (1176–1642)* SG	43 (39–48)* SG	20 (18–22)* SG		37 (33–41)* SG	-	-	14 (8–19)* SG
		60	-	1625 (1249–2002)* SG	44 (37–51)* SG	18 (14–22)* SG		38 (31–45)* SG	-	-	8 (1–16)* SG

Abbreviations: *DR* dietary record, *EI* energy intake, *FFQ* food frequency questionnaire, *FR* food record, *EWL* excess weight loss, *RYBG* Roux-en-Y gastric bypass, *SG* sleeve gastrectomy, WL weight loss

Values are expressed as mean ± SD unless otherwise indicated

^*^Data expressed as median (IQR)

### Relationship Between Weight Loss and Dietary Intake

A specific objective of this review was to test if daily energy intake is a major determinant of weight loss in the included studies. Total energy intake (Total EI, kcal/d) and percentage of excess weight loss (EWL%) reduction were assessed in a meta-analysis. Five studies were included in the subgroup ≥ 50 EWL% and < 75 EWL%, comprising a total of 211 patients. For this subgroup, at a 12-month follow-up, there was an average reduction of 861 kcal/d in a before and after analysis of RYGB (MD =  − 861, 95% CI − 1324 to − 398; *I*^2^ = 98%).

**Fig. 4:**
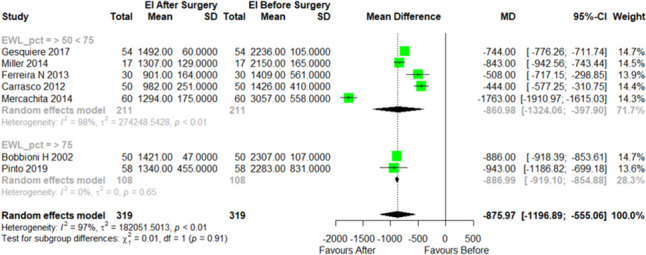
The association between energy intake (kcal/day) both before and after RYGB surgery and the percentage of excess weight loss (EWL%) 12-month post-surgery, including subgroup analysis (Number of studies = 7). SD = standard deviation; CI = confidence interval; pct = %. A successful outcome is defined as achieving ≥ 75% of EWL, an acceptable outcome falls within the range of 50% to 75% of EWL, and an unsuccessful outcome is indicated by < 50% of EWL

In the analysis of a second subgroup, including two studies and a total of 108 patients with EWL% ≥ 75%, the average reduction in Total EI was 887 kcal/d in a before and after RYGB analysis (MD =  − 887, 95% CI − 919 to − 855; *I*^2^ = 0%). Figure 4 illustrates the association between energy intake (kcal/day) before and after RYGB surgery and the EWL% 12-month post-surgery.

In the overall analysis, there was no difference in the reduction of total energy consumption between the two subgroups (test for subgroup differences (random effects model, *p* = 0.91)). These results show that there is no association between total energy intake (kcal/day) before and after RYGB surgery and the EWL% at 12-month post-surgery.

## Discussion

This review reports on the impact of bariatric surgery, both RYGB and SG, on total energy, and macro- and micro-nutrient intake. The findings indicate that there are no significant differences in energy intake, including energy and macronutrient intake, between RYGB and SG. Twelve months post-bariatric surgery, the proportion of carbohydrate intakes was reported to range between 35 and 53% of total energy intake, which largely aligns with AMDRs (45–65%). Protein intake was reported to range between 10 and 52%, suggesting that some patients have higher protein intake than AMDRs (10–35%). However, protein intake after bariatric surgery is generally found to fall below the recommended intake [20]. The American Society for Metabolic and Bariatric Surgery guidelines suggest that the daily protein intake should be between 60 and 120 g (1–1.5 per kilogram of desirable body weight) after surgery [55]. At 6-month post-surgery, we found that the reported average daily protein intake ranged widely from a minimum of 19.5 g/day (below recommended intake) to a maximum of 101.5 g/day (within recommended levels of intake).

Similarly, the daily fat intake ranged from 28 to 40%, which is slightly higher than the recommended AMDRs (25–35%). Micronutrient intake of zinc and iron appears lower than the recommended daily intake levels after surgery. Remarkably, and contrary to widespread assumptions, we found no relationship between average total daily energy intake and the degree of excess weight loss, or BMI achieved 12-month post-surgery.

More studies compared RYGB with gastric banding than with SG [55–58]; reflecting the relatively more recent introduction of SG in clinical practice [59]. Moreover, of the studies that compared RYGB with SG, the majority investigated eating behaviors rather than dietary intake [60–63]. Only seven studies compared dietary intake between RYGB and SG [8, 14–16, 24, 25, 48]. The most robust among these studies was conducted by Moizé and colleagues and investigated the changes in dietary intake over 5 years of post-surgery in a Mediterranean population. The authors found no difference between RYGB and SG in dietary intake. The daily dietary intake of micronutrients, including calcium, magnesium, phosphorus, and iron, was lower than the RDA for both types of surgery. However, a significant limitation of this study was its uneven distribution of participants between the two groups (RYGB, *n* = 294 versus SG, *n* = 61) [14].

Common limitations of studies comparing dietary intake between RYGB and SG were the short-term follow-up period and the paucity/absence of micronutrient intake data. Furthermore, energy intake in relation to weight loss may be influenced by physical activity [56], and this was not addressed in any of these studies.

There were 35 out of 42 studies reviewed here that examined the impact of RYGB and SG independently on dietary intake. The number of independent descriptive studies is higher than comparative studies on dietary intake after RYGB and SG. In addition, there are more descriptive studies on RYGB than on SG, especially in relation to the long-term outcomes.

In this systematic review, only 11 out of 42 studies investigated the micronutrient intake following RYGB and/or SG. In the short-term (≤ 12 months), vitamin B12 intakes met the RDA while zinc intake was less than 8 mg/day. In the longer term (> 12 months), vitamin B12 and zinc intakes were in line with the RDAs, but iron intake appeared to remain suboptimal at 20-month post-surgery. There are many studies on micronutrient status, however, far fewer studies are available on the micronutrient intake following RYGB and/or SG. Therefore, more studies are needed to further investigate the impact of bariatric surgery on micronutrient intake.

Much of the available evidence from studies that investigated dietary intake in relation to weight loss maintenance suggests that energy intake is not associated with long-term maintenance of weight loss following bariatric surgery [18, 31, 42, 45, 57]. In this systematic review, we have found no relationship between energy intake and weight loss after bariatric surgery. The finding that energy intake is not related to the weight loss outcomes of bariatric surgery is in contrast with the widespread belief that overeating is the cause of inadequate weight loss after bariatric surgery [46, 58–60].

Our review cannot make a firm conclusion on whether energy intake may contribute instead to long-term weight regain after surgery. Obesity is a multifactorial condition, and it is therefore plausible that resilient and/or recurrent pathophysiologic mechanisms may predispose individuals to disease recurrence. Bariatric surgery imposes substantial anatomical alterations to the GI tract [61]. Given the multiple metabolic and endocrine functions of the GI tract, changing the anatomy of the stomach and small intestine may affect energy homeostasis through several physiological mechanisms rather than merely due to mechanical restriction of energy intake [62]. Nutrient passage through the GI tract elicits secretion of several GI hormones (glucagon-like peptide-1, peptide YY, oxyntomodulin, GLP-2, glucose-dependent insulinotropic polypeptide, ghrelin) that play important roles in the regulation of hunger, satiety, and other metabolic functions [63–66]. In this context, the outcome of the interplay between the physiologic effects of GI surgery and pathophysiologic mechanisms of obesity is more likely to determine weight loss outcomes than either mechanical or voluntary restrictions of food intake.

Obesity is a complex, multifactorial medical condition caused by numerous factors, such as behavioral, psychological, biological, and social factors. Additionally, dietary intake is highly influenced by cultural background, socioeconomic setting, geographic environment, and product availability [67, 68]. These influencing factors were not considered in this systematic review, thus future systematic reviews should consider such an investigation. Additionally, methods used to assess dietary and nutrient intake are associated with known biases that can affect reporting of intakes, but this is a known limitation of dietary and nutrient assessment.

## Conclusions and Future Directions

In general, there is a paucity of comparative studies investigating the impact of RYGB and SG on dietary intake. Furthermore, most studies, both descriptive and comparative, investigated only short-term changes in energy intake and fewer data are available about the intake of micronutrients after bariatric surgery than macronutrients. Available data, however, do not support widespread assumptions that excess energy intake may explain poorer weight loss outcomes of bariatric surgery. While the role of energy intake on longer-term weight maintenance/weight regain needs to be further investigated, the results of this review suggest caution in attributing the efficacy of surgical treatment of obesity to a mere reduction of food intake.

More studies comparing nutrition intake after the two most performed bariatric procedures, especially in the long term, are needed. Further research is also necessary to understand the exact role of gastric versus intestinal anatomic manipulations in regulating post-operative energy intake and the role of energy intake as a mechanism of weight loss or metabolic control. More studies are also required to better assess the micro- and macro-nutrient intake after surgery using robust dietary assessment methods and to improve nutrition care strategies.
